# “We decided together”: a qualitative study about women with HIV navigating infant-feeding decisions with the father of their children

**DOI:** 10.1186/s12884-023-06198-w

**Published:** 2024-01-06

**Authors:** Bakita Kasadha, Shema Tariq, Nell Freeman-Romilly, Catherine Pope, Angelina Namiba, Farai Nyatsanza, Lisa Hinton, Tanvi Rai

**Affiliations:** 1 Nuffield Department of Primary Care Health Sciences, University of Oxford, Oxford, UK; 2 Institute for Global Health, University College London, London, UK; 3 Central and North West London NHS Foundation Trust, London, UK; 4 Oxford University Hospitals NHS Foundation Trust, Oxford, UK; 5 4M Mentor Mothers Network, London, UK; 6 Cambridgeshire Community Services NHS Trust, Cambridge, UK

**Keywords:** HIV prevention, Vertical transmission, Infant-feeding, Breastfeeding, Mother-to-child transmission, HIV

## Abstract

**Background:**

The World Health Organization (WHO) recommends that women with HIV breastfeed for a minimum of one year. In contrast, United Kingdom (UK) guidelines encourage formula feeding, but breastfeeding can be supported under certain circumstances. Infant-feeding decisions often involve personal and social networks. Currently, little research addresses how individuals with HIV in high-income countries navigate infant-feeding decisions with the father of their children.

**Methods:**

Semi-structured remote interviews were conducted with UK-based individuals with a confirmed HIV positive diagnosis who were pregnant or one-year postpartum, and two partners. Using purposive sampling, pregnant and postpartum participants were recruited through HIV NHS clinics and community-based organisations, and where possible, fathers were recruited via them. Data were analysed using thematic analysis and organised using NVivo 12.

**Results:**

Of the 36 women interviewed, 28 were postpartum. The majority were of Black African descent (*n* = 22) and born outside the UK. The key factors in women navigating HIV and infant-feeding discussions with respect to their baby’s father were the latter’s: (1) awareness of woman’s HIV status; (2) relationship with the woman; (3) confidence in infant-feeding decision; (4) support and opinion about woman’s infant-feeding intentions. Most women made a joint decision with biological fathers when in a long-term (> one year) relationship with them. Single women tended not to discuss their infant-feeding decision with the father of their child, often for safety reasons.

**Conclusion:**

Women in ongoing relationships with the father of their child valued their support and opinions regarding infant-feeding. In contrast, single women chose not to involve the father for reasons of privacy and safety. Clinical teams and community-based organisations should support mothers in discussing infant-feeding decisions regardless of relationship status. When appropriate, they should also support discussions with their partners, but remain sensitive to circumstances where this may put women at risk.

## Background

The World Health Organization (WHO) and United Nations Children’s Fund (UNICEF) guidelines on HIV and infant-feeding, strongly recommend breastfeeding for at least 12 months [1]. These guidelines are primarily intended for low-income settings where there is a more immediate risk of infant malnutrition or morbidity, through unsafe drinking water, than HIV transmission. Conversely, across several High Income Countries (HIC), including the UK, where consistent access to formula and safe drinking water is presumed, birthing parents living with HIV are advised to formula feed their babies exclusively [2–7]. However, in recent years, the UK HIV and infant-feeding guidelines from the British HIV Association (BHIVA) have been amended to state that individuals who choose to breastfeed can be supported to do so, as long as they are virologically suppressed and consent to additional blood monitoring of both themselves and their babies [2].

Data show that when on effective antiretroviral therapy (ART) with a fully suppressed HIV viral load, HIV cannot be transmitted via vaginal, oral, anal or any other type of sex, this is known as ‘Undetectable equals Untransmittable’ (U = U) [8]. However, while ART reduces the risk of infant acquisition of HIV significantly during breastfeeding, the risk is not zero. PROMISE, a multicentre randomised controlled trial, comparing maternal ART with infant nevirapine prophylaxis, reported breastmilk transmission rates of 0.3% and 0.6% after breastfeeding for six and 12 months respectively [9].

In the UK, between 600 and 1000 pregnancies occur in women with HIV annually [10], with low rates of vertical transmission (0.22%) [10, 11]. Around 65% of these women are Black African, and born in African nations [11]. There may be specific HIV-related stigma and negative consequences for African women in the diaspora who formula feed [12–14] especially in communities where formula feeding might signify an HIV-positive status [12, 15]. Generally, there are also strong personal and social ideals regarding motherhood and infant nourishment that are associated with breastfeeding [12, 14].

For these reasons, women living with HIV in high-income settings are increasingly considering breastfeeding as a safe, low risk option for infant-feeding although these numbers are still low [10, 16, 17]. Between 2012 and 2022, 203 women with HIV in the UK who had live births and were on ART with undetectable viral load, were reported to have been supported to breastfeed by their clinical teams [17]. In addition, some women with HIV may breastfeed without the knowledge or support of their clinical teams [16, 17].

Finding the balance between autonomy in infant-feeding decisions and adherence to national guidance is complex for those with HIV and the healthcare professionals advising and supporting them [2, 18]. As the UK guidelines become more supportive of breastfeeding we need a better understanding of the factors shaping parents’ decisions; this was the aim of our larger study (NOURISH-UK) [19]. In this paper, we focus on the role that fathers play in infant-feeding decisions in the context of HIV. There are currently limited data on this topic, with research predominantly focusing on cisgender women in isolation, removed from their wider social networks, or about fathers’ role but within a non-HIV context, or based in Low and Middle Income Countries (LMIC) settings. In research based in LMIC, HIV focused studies have highlighted that fathers can feel excluded from infant-feeding discussions due to gender norms or alienation from maternity healthcare services [20–25]. Additionally, due to the differing place-based infant-feeding policies mentioned earlier (i.e. breastfeeding being the official clinical policy in these settings), these studies primarily focus on the role of fathers in facilitating breastfeeding, rather than discussions and decisions about infant-feeding options more broadly. We have found no similar research from high income settings.

This paper explores how fathers influence infant-feeding decisions in the context of preventing vertical transmission of HIV. Drawing upon qualitative interviews conducted in the UK with pregnant women and mothers living with HIV, and two male partners, we explore the extent to which fathers were involved in infant-feeding decisions; and how these were shaped by wider contextual factors including relationship status, fathers’ own HIV status and views about HIV and stigma; and their socio-material circumstances.

## Methods

### Study design and recruitment

This paper is part of a larger study (NOURISH-UK, https://www.phc.ox.ac.uk/research/health-experiences/Nourish_UK) investigating infant-feeding decisions in the context of HIV in the UK. This was a qualitative study using semi-structured interviews. The interviews were conducted in two stages: first we conducted semi-structured interviews with women living with HIV who were either pregnant or postpartum at the time of interview. Following this, we also collected a much smaller set of semi-structured interviews with partners of the female participants.

Between April 2021 and January 2022, we recruited pregnant women and mothers living with HIV through the National Health Service (NHS) HIV clinics, HIV charities and community-based organisations, as well as via snowballing and personal networks. BK also joined mother and baby groups organised by three different HIV charities in order to promote the study.

Participants were eligible if they were 18 years-old or over, self-reported to be HIV positive, living in the UK and pregnant or had given birth within 12 months of the interview. Participants were selected through purposive sampling and we sought to achieve data saturation (i.e. no new categories can be added and no new major themes are emerging).

The study was originally intended for mothers and birthing parents alone. However, as the interviews progressed, it became apparent that partners may have an important role in the infant-feeding process so in consultation with the study team, patient and public involvement (PPI) contributors and the advisory panel, we decided to also include a small number of the women’s partners (*n* ≤ 5). Due to the nature of the study ethical approval (details below), a formal amendment was not needed for this, and we enlisted approval from the study funder to expand the sample in this way. In our sample, for all women in current relationships, their partners were also the biological fathers and therefore we have used the terms ‘fathers’ and ‘partners’ interchangeably.

### Interview topic guide

The topic guide for the larger NOURISH-UK study was written by BK and TR, and developed with the study team and members of the PPI panel. All members of the PPI panel were mothers living with HIV.

We had different topic guides for women with HIV and their partners. The former covered experiences of being diagnosed with HIV, their intimate and personal relationships, social networks, feelings about parenthood including experiences of having older children, their understanding of infant-feeding options, the national guidelines and healthcare experiences, and role of their baby’s father in the infant feeding decision. Participants were also asked if they were aware of the HIV status of the father of their child; if the HIV status was known, we learned if it had been a serosame (both partners living with HIV) or serodifferent (the father was HIV-negative) coupling.

The topic guide for the partners of our initial participants covered many of the above themes, as well as their own HIV status (if known), their attitudes towards their partners’ HIV status and breastfeeding, and their knowledge of HIV and infant-feeding, and the associated guidelines.

Brief demographic data were captured for both kinds of participants, based on recommendations for gender reporting in order to capture transgender status of participants (where applicable) [11] and we used national ethnic demographic categories [26].

### Data collection

BK (field researcher) and TR (principal investigator), both women from racially-minoritised groups and within their respective academic roles, conducted the qualitative semi-structured interviews. Interviews were held remotely (due to the COVID-19 pandemic), either via telephone or MS Teams. All participants were interviewed once. Both interviewers made field notes during and immediately following the interview.

Prior to each interview, the interviewer arranged a pre-interview introductory conversation about the study, as well as answer any queries the prospective interviewees had. They were then sent the study information sheet via post or email. For those who consented to be contacted again, interviews were scheduled at least one week after the introductory call.

This study had a two-staged process for taking informed consent: participants provided verbal consent to be interviewed; post-interview they provided written consent for their data to be included in the study (including, but not exclusive to, analysis and dissemination). The two-step informed consent process is part of our study protocol as part of our dissemination includes a public facing website (https://healthtalk.org/Feeding-a-baby-while-living-with-HIV/overview). Each interview lasted approximately one hour and all were audio-recorded and transcribed verbatim. Each participant received a £20 shopping voucher.

### Data analysis

BK and TR analysed the data using thematic methods [27], incorporating a mind-mapping approach one sheet of paper (OSOP) [28] to support critical, reflective analysis. Both inductive and deductive thematic analyses were used, through which BK and TR developed a coding framework which was applied iteratively to the data. They grouped related extracts from interview transcripts around themes, which were then analysed further using mind-maps that they developed independently, and then discussed together, to resolve any differences. The OSOP mind mapping method [26] enables all of the relevant data to be included in the thematic analysis, and is a thorough and auditable approach. We have provided a distilled version of the common themes and sub-themes below (Table 3).

### Stakeholder engagement

Stakeholder engagement was central to the NOURISH-UK study. Our study team and the advisory panel included mothers living with HIV alongside representatives from community based organisations and healthcare professionals working in HIV and/or antenatal care [29]. Our Patient Public and Involvement (PPI) panel sat within the broader study advisory panel and was comprised of five women with experience of pregnancy and motherhood while living with HIV, as well as experience of supporting other mothers living with HIV [30]. The PPI lead was also part of the study team and a named co-applicant.

### Ethical approval

Ethics approval for the NOURISH-UK project (including the extension to include interviewing fathers) is in place under the long-term study ‘Narratives of Health and Illness for www.healthtalk.org’. This study is approved by NRES Committee South Central –Berkshire (12/SC/0495), and the HRA and is included on the National Institute for Health Research Network (NIHR CRN) Portfolio (IRAS Ref: 112111. Study ID: 13550).

## Results

Of the 45 individuals who provided verbal consent to be interviewed, 38 participants provided post-interview written consent to be included in this study. Our sample included 36 cisgender women living with HIV (eight pregnant and 28 postpartum) and two male partners (Tables 1 and 2). Twenty-eight women were in a relationship with the father of their children. The majority of women who were postpartum had an undetectable HIV viral load at the time of birth (*n* = 26). Twenty of the 36 women interviewed postpartum had formula fed; none of our participants reported vertical transmission of HIV to their infants. Of the women who were pregnant at the time of interview, four intended to breastfeed, two planned to formula feed and two were undecided. One woman had a six-month old baby and was pregnant, therefore the interview data referred to current and planned formula feeding decisions. Of the 28 women who were in relationships (all with a duration of over one year), seven women who reported being in serosame status relationships and two reported that their long-term partner was unaware of their HIV status. Neither of the male partners we interviewed had HIV themselves (Table 2), and both were aware of their partners’ HIV status.

**Table 1:** Women’s characteristics

**Pregnant women and mothers (*n* = 36)**	
**Characteristic**	**N**
**Age (years)**	
18 – 24	2
25—29	6
30 – 34	8
35—39	10
40—44	10
**Ethnicity (self-described)**	
Asian	3
Black African	22
Black Caribbean	2
White British	6
White other	2
Not known / stated	1
**Place of birth**	
Africa	21
Mainland Europe	2
UK and Ireland	11
Elsewhere	2
**Diagnosed during most recent pregnancy**	
Yes	5
No	31
**Timing of HIV diagnosis**	
< 1 year	2
1—10 years	22
11 to 25 years	9
26 + years^a^	3
**Partner’s HIV status (where in a relationship, *n* = 28)**	
HIV-positive	7
HIV-negative	18
Not known / stated	3
**Basic needs met**	
All / most the time	16
Some/ none of the time	16
No answer	4

^a^Pre effective ART

**Table 2:** Male partners’ characteristics

Male partners (*n* = 2)	
**Age (years)**	
30 – 34	1
35—39	1
**Ethnicity (self-described)**	
White British	2
**Country of birth**	
UK	2
**HIV status**	
HIV negative	2
**Basic needs met**	
All of the time	2

Table 3 provides an overview of the common thematic subjects and sub-themes. The analysis presented in this paper focuses on interview extracts pertaining to women’s accounts of the roles the father of their children played in infant decision making. Data files were managed using NVivo 12. Throughout this paper, we present illustrative quotes, with pseudonyms chosen by participants.

**Table 3:** Common thematic subjects and sub-themes

**Common thematic subjects**	**Sub-themes**
**Father’s awareness of woman’s HIV status**	Confidence in/experience of sharing HIV status with father of child
	Father's medical literacy and knowledge of HIV
	Father's HIV status
**Father’s relationship with the woman**	Relationship status
	Threats of disclosure and domestic abuse
	Gendered roles and expectations regarding infant-feeding decision making
**Father’s confidence in infant-feeding decision**	Woman’s medical literacy to communicate infant-feeding guidance and knowledge
	Father's medical literacy to understand infant-feeding guidance
	Father's pre-existing infant-feeding preferences
	Conversations with healthcare professionals
**Father’s support and opinion about woman’s infant-feeding intentions**	Joint decision making
	Woman leading decision making
	Father leading decision making
	Conflict management and resolutions

Due to the interconnectedness between the sub-themes and to maintain the flow of the narrative, we have structured our results based on the common thematic subjects.

We structured our results around four themes linked to father’s: (1) awareness of woman’s HIV status; (2) relationship with the woman; (3) confidence in infant-feeding decision; and (4) support and opinion about woman’s infant-feeding intentions.

- 1. Father’s awareness of woman’s HIV status

Of the 28 women who were in relationships (all with a duration of over one year), two reported that their long-term partner was unaware of their HIV status. Women in relationships reported that their partners’ knowledge of their HIV status had facilitated joint and informed decision-making.

“We both attended that appointment. It was nice for us to both hear the same information, wasn’t me telling him [...] [we] just wanted the same outcome whatever is the safest option in terms of for our baby, not going to give him like slightly chance of getting it […]that was one of our main concerns.”

- Maya, 27yo, serodifferent relationship, 11-month-old baby, formula fed

“He definitely was [involved] in both situations [pregnancies], he was very much like ‘I think we do the kind of lowest risk route possible for the babies’ and that was the formula [milk] then, he said as long as that’s what I wanted to do […] It was never like ‘this is what we’re doing kind of thing’, it was more of a, you know, ‘are we both happy with that […] we were both in the same boat I guess so we were both, ‘yeah, I think we’re okay with this.’”

- Sinead, 42yo, serosame relationship, nine-month-old baby, formula fed

However, one participant reported that, although her husband was aware of her HIV status, he was not aware that UK national guidelines recommended formula feeding. She knew that he favoured breastfeeding, and felt ‘relief’ when her doctor informed her that breastfeeding was an option.

“when I’d kind of spoken to my husband about the [HIV] diagnosis I hadn’t really mentioned to him that ‘Oh I wouldn’t be able to breastfeed’ […] he’s always been so heavy on the breastfeeding side of things, so when, when my second doctor had said ‘oh yeah you can breastfeed’ and I was like it was just such a relief […] because I don’t have to face that kind of conversation with my husband as well […] Just like he’s read about the benefits of it [breastfeeding] and his friends’ wives and so they’ve all breastfed […] he just knows as well that it’s just best for the baby”

- Maria, 37yo, serodifferent relationship, 16-month-old baby, breastfed

A minority of women reported that they had had general conversations about infant-feeding with their partners, but not specifically about the HIV transmission risk associated with each feeding option. Women did not involve their partners if, for example, they were not aware of the woman’s HIV status, as was the case for two women. Biola shared that “The dad of my children, he don’t know about my status at all” and her sole reason for breastfeeding was to avoid signalling her HIV status to her partner.

“So it’s hard for me to tell him that I’m HIV. The moment [you do] you might fight in the street.”

- Biola, 39yo, serodifferent relationship, eight-month-old baby, breastfed

Another participant (Nozipho) reported that her partner not knowing her HIV status had contributed to her desire to breastfeed, however it was not the main driver. She wondered whether her partner already knew about her HIV status (because her HIV medication was kept visible in the home). She was primarily motivated to breastfeed because of the bonding and nutritional benefits for the baby. When asked if her partner being unaware of her HIV status had influenced her decision to breastfeed, she replied:

“ [...] that’s what the midwives thought, but to be honest I’ve known this guy since we were in our early twenties […] let’s just say maybe, out of 100, 20%, or 25% yes [him finding out] was at the back of my mind.”

- Nozipho, 30yo, serodifferent relationship, 11-month-old baby, formula fed

Therefore when women reported that the father of their child was aware of their own HIV status, they felt able to engage fathers in joint decision making about their infant-feeding options.

- 2. Father’s relationship with the woman

In general, women in relationships with the father of the child (when fathers were aware of the women’s HIV status) involved their partners in their infant-feeding decision. Just one woman (Amina, quoted later) reported that her husband (also living with HIV) was not involved because he believed that only mothers should make infant-feeding decisions.

In contrast, the majority of the unpartnered women we spoke to did not discuss their decision with the father of their child. April, who suspected that her ex-husband was also HIV positive, was single by the time she gave birth and said:

“No, no, no I did not discuss it with him. I made my own decision […] we had our problems like for a very long period of time […] and by the time I got pregnant I was in the process of leaving him […] when I was pregnant I was on my own all this time for the nine months so I did not include him in making decisions.”

- April, 40yo, single, four-month-old baby, formula fed

For some single women, the threat or knowledge of their HIV status being shared and/or intimate partner violence (IPV) constrained open discussion with the father of their children. Kay, originally from the US, reported that the father of her child (based in the US) was not HIV positive, and had been threatening her since she got pregnant. She planned to give birth in the UK because of more supportive breastfeeding policies and laws (compared to the US).

“the hostility between the father of the child and I, like he has made comments that, about what he could bring up in court which I think like my status as perhaps an implied measure and then he says well we should talk about things we agree not to bring up in court which again I always feel like [he’s] very heavily implying my [HIV] status […] I asked my medical professionals like if someone tries to bring this up in court that I’m an unfit mother that I’m HIV positive and that I’m breastfeeding my child […] They said their reaction to that was ‘well we want you to know that we would come testify as your medical team, that we’re supporting you in making this decision [to breastfeed]’”

- Kay, 31yo, single, pregnant, intends to breastfeed

Women’s relationship status and proximity to the father of their child held great importance in determining their comfort discussing infant-feeding options with them, regardless of the fathers’ own HIV status.

- 3. Father’s confidence in infant-feeding decision

The two fathers interviewed were not living with HIV themselves but were aware of their respective partner’s HIV status. One reported that his wife’s work for an HIV community-based organisation gave him confidence in her infant-feeding knowledge and decision, especially considering the infrequency of clinical appointments.

“I feel that if it wasn’t for her knowledge of [breastfeeding] I think it would be a very different story […] I don’t feel like the conversation would have gone the same way. I feel like the appointments with consultants and stuff are so few and far between that it doesn’t […] I almost feel like you’re too far down the road before that conversation’s even started to happen and you could [have] already made a decision or if you have made a decision and then you start to learn about it, it makes that decision even harder when I don’t think it should. I think the information should be made perhaps a bit clearer at the start.”

- Edward, 32yo, wife is pregnant

As mentioned previously, one woman said that her husband (also living with HIV) knew her HIV status and was happy for her to make infant-feeding decisions on her own:

“He knows that I’m like I’m good to make decisions [...] he’s just like, “Do what’s best for you.” […] it’s not his place to say I guess so […] I’m free to choose or choose not to.”

- Amina, 23yo, serosame relationship, pregnant, intends to formula feed

Overall, how father’s viewed their partner’s ability to gather and discern key information, impacted the trust they had in women leading the infant-feeding decision. As is the case with maternity journeys in general, our women participants felt closer to the infant-feeding decisions since they were bearing the child, and had encountered more (clinical and non-clinical) information and support than the fathers.

- 4. Father’s support and opinion about woman’s infant-feeding intentions

As mentioned before, the majority of women in relationships with the fathers of their children emphasised that it had been a joint decision. Despite knowledge of the guidelines and a strong desire to breastfeed, Marella relied on the support of her partner:

“[I]f they [my husband, mum or HIV physician] had shut me down […] said it [breastfeeding] is not a good idea, I don’t think I’d be as confident as I am now.”

- Marella, 30yo, serodifferent relationship, pregnant, intends to breastfeed

Likewise, Holly mentioned how vital her partner’s support was:

“[Women with HIV need to] be really confident perhaps of your support network and […] the man that is involved in your life. I think I couldn’t do it without my partner”

- Holly, 36yo, serodifferent relationship, pregnant, intends to breastfeed

A minority of women reported being swayed by their partner’s preference. For example, Layla decided not to breastfeed because of her husband’s concerns:

“Well I actually wanted to breastfeed more than my husband but he was very much like yeah there’s a risk don’t do it”

- Layla, 35yo, partnered, serodifferent relationship, 11-month-old baby, formula fed

In contrast, Stephen described being more in favour of breastfeeding than his pregnant partner and how attending antenatal classes had influenced her infant-feeding decision:

“I’m more pro-breastfeeding whereas [my partner] is not so pro […] I think she’s just more scared about the risk of breastfeeding and her mum didn’t breastfeed her so she’s like ‘well I turned into a strong adult.’ […] we’ve been doing NCT [antenatal] classes and they have midwives who are pushing breastfeeding and kind of like giving you the benefits and kind of yeah this is what you should be doing. So now I think her mind’s slightly changed about it.”

- Stephen, 37yo, partner is pregnant

As it was rare for couples to have opposing views, generally partners were able to either make joint infant-feeding decisions or women took the lead in suggesting their infant-feeding preference.

## Discussion

The NOURISH-UK study aimed to understand how pregnant and postpartum individuals living with HIV in the UK make decisions about infant-feeding. In this paper, we have presented what we believe is the first qualitative investigation of how women living with HIV navigate infant-feeding decisions with the father of their children in High Income Countries (HIC). We found that women’s decisions about how they feed their infants are not made by themselves alone, and that partners often play a critical role. Relationship status (at the time of interview) and partner’s knowledge of their HIV status were both important factors influencing whether women discuss their infant-feeding decision with the father of their children. The women in our study who were in a relationship with the father of their baby, tended to consider their partners’ opinions and support to be important and many had made joint decisions. Only one woman reported being discouraged from breastfeeding by her partner because of his concerns about transmission risk. Generally, where partners were aware of their HIV status, women did not identify HIV-stigma as a barrier to discussions about infant-feeding choices. This was also reflected in the accounts of the two male partners (neither living with HIV) who had valued being actively involved in infant-feeding decisions with their partners. Aside from two female participants in relationships where their partner was unaware of their HIV status, the majority of those in relationships had experienced joint decision-making and negotiation.

The duality of relationship status and partners knowledge of HIV status were important contributors in determining how our female participants involved their partners in their infant-feeding decision. Women who had separated from the fathers of their children did not discuss infant-feeding with them due to being estranged, and some had concerns about ex-partners disclosing their HIV status to others, or felt at risk of intimate partner violence (IPV). Nine of the women we spoke with (including seven of the partnered women), reported that the father of their children was also HIV positive. However, it was beyond the scope of this study to investigate the strength and openness of these relationships and how these contributed to the ease or difficulty of instigating these conversations. Nonetheless, this study provides novel insights, as there is little research in contexts where the risk of women’s HIV status being disclosed to the partner is not an issue. Moreover, our data showed that fear of signalling one’s HIV status (when the father was HIV-negative) became less influential in the infant-feeding decision for women who were now separated from the father. These women’s reasons for wanting to either formula or breastfeed were multifaceted and based on risk of transmission to the infant versus the benefits of breastfeeding to mother and baby.

Many of our findings contrasts with previous literature (which importantly, is not based on the HIV context) and highlight that current clinical practice may overlook the role of fathers in the infant-feeding decision. In the non-HIV literature, social support, particularly from partners, is linked to improved clinical and other pregnancy outcomes [31–33]. However other studies, also outside the context of HIV, found that men struggle to attend antenatal visits and engage in antenatal conversations due to structural and local cultural norms about gender roles and responsibilities, which can result in them not contributing to the decision-making process [34–38]. We know that in the general population, fathers’ attitudes towards infant-feeding are largely influenced by their families, partners and/or healthcare professionals [39]. However, these studies largely explored fathers’ role in facilitating and *supporting breastfeeding*, especially studies from Low and Middle Income Countries (LMIC). This situation is quite different to our research which has investigated fathers’ role in the actual decision regarding *how* to feed babies, especially when it is a complex one due to a context where formula feeding is the clinically-recommended standard, and there are real (albeit small) infant health risks associated with breastfeeding (i.e. HIV transmission). Existing research on the role of fathers’ support in facilitating exclusive breastfeeding reveals mixed findings on fathers’ enthusiasm or confidence in getting involved, even in countries where encouraging paternal involvement is part of national policy [3, 13, 40]. However, our data reveals that fathers (who were also partnered to the mother of their child) held an important and active role in the infant-feeding decision.

Our unpartnered participants’ fears of or experiences of IPV supports wider literature that women with HIV are disproportionately affected by domestic abuse [41–45]. The pregnancy and the postpartum periods are recognised as particularly high risk times for women in terms of intimate partner violence [46, 47]. Previous studies (non-HIV related) have established a correlation between maternal stress, depressive symptoms and anxiety, with an increase level of maternal stress between pregnancy and post-partum [48]; this would be made worse by the acute stress of living with an HIV diagnosis, having to consider risk of vertical transmission and/or accidentally disclosing one’s HIV status [12]. Having a supportive partner during this intense period, who does not hold HIV-stigma and is able to engage in the complexity of the infant-feeding decision was a source of strength for many of our participants.

### Strengths and limitations

This is the first qualitative UK-based study since the British HIV and infant-feeding guidelines changed in 2018, our findings suggest that infant-feeding conversations may be less fraught than in the past, possibly due to these more nuanced and supportive guidelines. However, we acknowledge that our sample was a self-selecting group and we did not specifically ask how long each participant had been in their relationship, which may have impacted comfort levels regarding sharing one’s HIV status and discussing infant-feeding decisions. We interviewed two fathers (neither living with HIV themselves) therefore experiences of fathers were largely reported by the women in this study; the fathers themselves may have had different perspectives. Accounts regarding risk of HIV disclosure and IPV were rare in this cohort and may have been due to selection bias or underreporting due to HIV stigma and fears over transmission (vertical or otherwise), as well as existing UK criminalisation and safeguarding policies [45, 49–54].

All interviews were held online due to COVID-19 restrictions. The majority of participants were alone at the time of their interview, however some were accompanied by their infants and a minority had their partners present which may have influenced their responses. As more data collection takes place online, privacy and possible interruptions should be anticipated by all researchers collecting data virtually [55]. Prior to the interview all participants were sent a document on how to maintain privacy and confidentiality during an online interview and were given the option to pause or hold the interview over different days. Despite efforts, we were unable to recruit gender diverse or sexually minoritised people to our study. Most of our participants were of African descent and we had a small number of women of South Asian heritage and White women—this was reflective of the population of women with HIV in the UK.

### Implications for clinical practice and research

These findings have wider implications for research and clinical practice on the role of fathers in infant-feeding in HIV. The majority of women reported that their partners’ opinions were informed by national guidelines, wider (non-HIV specific) breastfeeding policies, as well as the opinion of the women themselves. Therefore, UK guidelines on pregnancy and infant-feeding in HIV should explicitly address the potential role of fathers in infant-feeding decisions and encourage multidisciplinary teams to include fathers in discussions when safe and appropriate (particularly when they are in a relationship with the mother of their children). Tailored online and offline patient information targeting fathers may support this [56], keeping in mind that fathers of children born to women with HIV are a heterogeneous group, and will include men who may be living with HIV themselves with varying degrees of HIV knowledge. The content and delivery of information need to take into account these differences.

Healthcare professionals should also be aware that in some situations there may be potential risks to women and their babies by involving partners (such as IPV or HIV status disclosure). As with the guidelines and information materials, multidisciplinary teams should assess what support women and birthing parents may need in deciding whether to inform their partners of their HIV status and navigate discussions about infant-feeding decisions and the quality of the partner relationship. Relationship status and the quality of the relationship may impact how women may wish to involve the father of their children in infant-feeding conversations. Healthcare professionals may need to facilitate conversations and address knowledge gaps within couples, for example, where the partner may be aware they are in a serodifferent relationship, but unaware of HIV and infant-feeding guidelines. Conversely, former partners may present a risk to women in terms of IPV and potential sharing of knowledge of HIV status. Multidisciplinary teams should ascertain women’s proximity to the father of their child, and (as already recommended in BHIVA pregnancy guidelines) continue to screen for possible IPV. Furthermore, to ensure equitable access, clear pathways to additional support are needed, such as HIV-peer support groups, which have been found to improve the confidence of parents considering these conversations with their partners, especially between clinical appointments [57].

Further research focusing on fathers or approaching couples as dyads (with biological and non-biological fathers) will help to further understand the information needs among fathers navigating and supporting infant-feeding decisions in the context of HIV. Our findings suggest that the nature of the relationships (i.e. healthy, unhealthy or abusive) may impact whether infant-feeding is discussed by women with the father of their children, and the transparency of these discussions. Additionally, research on gender diverse and non-heterosexual parents would help to identify the role of gender and sexuality in infant-feeding conversations with partners.

## Conclusion

Fathers have an important role in infant-feeding decisions within the context of HIV, when they are also partnered with the mother of their children. Within this context, fathers are influenced by their partners’ feeding preference and access to robust information about the latest HIV and infant-feeding guidance, however they may need tailored information and support to be confident in their infant-feeding discussions and decisions. In contrast, unpartnered women tend not to rely on the father of their children when making infant-feeding decisions and, due to privacy and safety concerns, and may actively exclude them from these conversations. Regardless of relationship status, pregnant women and mothers living with HIV depend on clinical teams’ support to navigate their overall infant-feeding decisions. These professionals may be an additional source of support for women who are considering discussing their HIV status or the complexities around HIV and infant-feeding with the father of their children, or may offer support in the circumstances where it may be less safe for women to involve the father of their children.

## Terminology

Throughout the study, including within this paper we use non-stigmatising language, including person centred and person first language [58–62]. We use the term ‘vertical transmission’ to refer to HIV transmission to a baby in utero, childbirth or while breastfeeding. We intentionally avoid the use of ‘mother-to-child transmission’. Although this study sought to be transgender inclusive, we were only contacted by cisgender women, therefore we use ‘mother’ and ‘pregnant women’ when discussing our data, with the understanding that birthing parents living with HIV of other genders may share similar experiences.

## Data Availability

The healthtalk.org *Feeding a baby while living with HIV* web resource may aid conversations between HCPs, women and their partners (https://healthtalk.org/Feeding-a-baby-while-living-with-HIV/overview) and includes additional data from this study. All participants and/or their legal guardian(s) provided informed consent for publication of any identifying information/images contained on this website. The datasets used and/or analysed during the current study available from the corresponding author on reasonable request.

## Abbreviations

ART: Antiretroviral therapy
HCP: Healthcare professionals
HIV: Human immunodeficiency virus
MS Teams: Microsoft Teams
NHS: National Health Service
PIS: Participant information sheet
PPI: Public and patient involvement
UK: United Kingdom
US: United States
UNICEF: United Nations Children’s Fund
WHO: World Health Organization
